# Combined effects of Yupingfeng and metformin on blood glucose levels and intestinal tissue in high-fat diet-induced type 2 diabetic rats

**DOI:** 10.3389/fendo.2025.1532560

**Published:** 2025-07-23

**Authors:** Hui Liu, Yuan Guan, Like Xu, Ji Hu

**Affiliations:** ^1^ Medical College, Suzhou University, SuZhou, Jiangsu, China; ^2^ Affiliated First People’s Hospital of Endocrinology, Yangzhou University, Yangzhou, Jiangsu, China; ^3^ The Affiliated Hospital of Yangzhou University, Yangzhou University, Yangzhou, Jiangsu, China; ^4^ The Second Affiliated Hospital of Suzhou University, 1055 Sanxiang Road, Suzhou, Jiangsu, China

**Keywords:** type 2 diabetes models, blood sugar index, intestinal inflammation, combination therapy, SD rat

## Abstract

This study investigated the effectiveness of a high-fat diet (HFD) in inducing type 2 diabetes in Sprague–Dawley (SD) rats and evaluated the combined therapeutic effects of Yupingfeng and metformin on glycemic index and intestinal histology. A total of 60 SD rats, equally divided by sex, were randomly allocated to six groups. Body weight was measured weekly, and food intake was recorded. During the initial phase of model induction, blood glucose levels increased significantly, exceeding 17.26 mmol/L by week 8, which was significantly higher than that of the control group (*p*< 0.01). The experimental group received treatment for a total of 7 weeks. Beginning in the third week, the combined administration of Yupingfeng and metformin significantly reduced fasting insulin levels, blood glucose concentrations, and insulin resistance compared to the control group, with further marked reductions observed by the seventh week (*p*< 0.01). Lipid metabolism indicators, including triglycerides (TG), total cholesterol (TCHO), high-density lipoprotein cholesterol (HDL-c), and low-density lipoprotein cholesterol (LDL-c), showed significant reductions in the treatment groups (*p*< 0.01). Liver and kidney function assessments showed that the combination therapy exhibited superior safety compared to single-drug treatments. In addition, hematoxylin and eosin (H&E) staining revealed that HFD-induced diabetes caused inflammatory changes in the duodenum and colon. However, the combination therapy markedly alleviated inflammatory symptoms. Overall, the combined treatment significantly improved blood glucose levels, biochemical parameters, and intestinal tissue integrity.

## 1 Introduction

A high-fat diet (HFD) is a major contributor to elevated blood glucose levels, which plays a key role in the development of type 2 diabetes (T2D). Epidemiological data indicate that approximately 90% of the estimated 537 million diabetes cases worldwide are classified as type 2. Alarmingly, the prevalence of T2D is increasing rapidly, particularly among children and young adults under the age of 40 (1). Research in Western Ethiopia has shown a strong correlation between the physical condition and quality of life of patients with T2D during rehabilitation. Lifestyle and dietary changes have contributed to the continued increase in T2D cases, with a growing trend among younger individuals, especially those under 40 (2). Male patients with T2D outnumber their female counterparts, and the condition can lead to various complications, including retinopathy, stroke, coronary artery disease, kidney disease, peripheral artery disease, peripheral neuropathy, and gastrointestinal disorders such as ulcerative gastritis and ulcerative colitis, all closely linked to high blood glucose levels (3). Clinically, a variety of antidiabetic medications are available both domestically and internationally. Among the most commonly prescribed are metformin and α-glucosidase inhibitors, which help to regulate key metabolic and inflammatory parameters such as fasting plasma glucose (FPG), fasting insulin (FINS), glycated hemoglobin (HbA1c), and homeostasis model assessment of insulin resistance (HOMA-IR). These agents also influence inflammatory cytokines like tumor necrosis factor-alpha (TNF-α), interleukin-6 (IL-6), and adiponectin (APN), as well as markers for lipid metabolism [total cholesterol (TCHO), triglycerides (TG), and low-density lipoprotein cholesterol (LDL-c)], and liver function indicators [alanine aminotransferase (ALT), aspartate aminotransferase (AST), and gamma-glutamyl transferase (GGT)]. However, despite these pharmacological options, the success rate in achieving optimal therapeutic outcomes remains disappointingly low, with less than 50% of patients reaching targeted treatment goals (4). Consequently, the development of more effective and targeted therapies for T2D continues to be a major area of research and clinical interest.

In 2019, the prevalence of T2D in China reached 14.92%, making it the country with the highest number of patients with diabetes worldwide, and accounting for approximately one-quarter of global cases (5). A study conducted in Western Ethiopia found a strong association between physical wellbeing and quality of life in patients with T2D during rehabilitation. The rising incidence of T2D, particularly among younger individuals, is largely attributed to changes in lifestyle and dietary habits (6). Emerging research has also highlighted the role of gut microbiota and natural compounds in metabolic regulation. For instance, a study demonstrated that 4 weeks of fecal microbiota transplantation significantly improved glucose tolerance in diet-induced obese mice by activating colonic bile acid receptors, enhancing host immune-inflammatory responses, and increasing circulating glucagon-like peptide-1 (GLP-1) concentrations (7). Similarly, quercetin—an active flavonoid extracted from vegetables and fruits—was shown to improve metabolic function and reduce organ damage in both streptozotocin (STZ)-induced and genetically inherited mouse models of T2D (8). Given these findings, the integration of conventional antidiabetic drugs with traditional Chinese medicine (TCM) is now a major focus of current research aimed at reducing glycemic index of T2D and minimize diabetes-related organ damage more effectively

Yupingfeng is a traditional Chinese herb primarily composed of Huangqi (*Astragalus membranaceus*), known for its properties in strengthening the exterior (to make the skin firm), controlling perspiration, nourishing the lungs, and boosting qi (i.e., vital energy for life) (9). Fangfeng (*Saposhnikovia divaricata*) serves as an auxiliary herb that addresses muscle-related discomfort and dispels pathogenic wind. In clinical applications, Yupingfeng is formulated into granules by combining dextrin, mannitol, flavor-enhancing agents, and pharmaceutical binders to facilitate oral administration (10). Currently, Yupingfeng granules are widely used to prevent and treat common colds, reduce inflammatory responses, and manage vascular and allergic conditions, particularly allergic rhinitis. The formulation is recognized for its therapeutic efficacy, safety, and minimal side effects (11). A systematic review of randomized controlled trials (RCTs), covering literature up to 28 November 2018, found that Yupingfeng significantly improved respiratory symptoms in patients with pediatric asthma, reducing cough, wheezing episodes, and the frequency of asthma attacks (12). Furthermore, studies in BALB/c mice have demonstrated that Yupingfeng powder possesses antifungal properties, significantly inhibiting the growth of *Candida albicans*. It also reduced infection-induced renal inflammation, improved the CD4/CD8 T-cell ratio, and activated the IL-17 signaling pathway in spleen lymphocytes, collectively enhancing immune function (13). Despite its wide range of traditional and experimental applications, there are currently no domestic or international studies exploring the potential of Yupingfeng powder in regulating blood glucose levels, improving blood biochemical markers associated with HFDs, or evaluating its effects on physiological and biochemical parameters in experimental models of metabolic disorders.

Metformin is the most commonly prescribed biguanide for lowering blood glucose levels in clinical practice. Extensive clinical data have demonstrated that metformin not only improves blood glucose levels in diabetic patients, but also offers cardiovascular protection, significantly reducing the risk of cardiovascular complications (14). In a study involving 445 overweight or obese Asian patents with T2D, with an average age of approximately 55.5 years and a disease duration of over 6 years, participants received metformin at doses of 5 and 15 mg. The treatment resulted in reductions in hemoglobin A1c (HbAlc) levels by 0.78% and 0.8%, respectively. Notably, it was established that metformin alone may not be sufficient to manage blood glucose in patients with T2D, but it can significantly contribute to weight reduction, a key factor in diabetes management (15). Further research found that combining metformin with sodium glucose cotransporter-2 (SGLT2) inhibitor can enhance therapeutic outcomes by lowering fasting blood glucose, increasing the urinary glucose-to-creatinine ratio, and significantly reducing body weight and blood pressure in clinical populations with T2D (16). Additionally, when compared to metformin, polyethylene glycol chloride has been found to improve several cardiovascular risk factors such as body weight, waist circumference, visceral fat area, blood pressure, and blood lipid profiles. It appears to have nearly equivalent effectiveness as metformin in controlling blood sugar levels (17). However, to date, no domestic or international studies have examined whether the combination of Yupingfeng and metformin can effectively reduce blood glucose levels, improve biochemical markers, or alleviate intestinal inflammation in models of T2D.

## 2 Materials and methods

### 2.1 Main reagents and instruments

The standard diet for Sprague–Dawley (SD) rats was supplied by Shanghai Slake Experimental Animal Co., Ltd., and consisted of 12% fat, 20.6% protein, and 67.4% carbohydrates. The HFD containing 45.1% fat, 20.1% protein, and 34.7% carbohydrates was obtained from Research Diets, Inc. (USA). Pharmaceutical agents were provided by Boehringer Ingelheim Pharma GmbH& Co. KG (Germany). The traditional Chinese herbal medicine “Yupingfeng,” composed of 30 g of Fangfeng, 60 g of Huangqi, and 60 g of *Atractylodes macrocephala*, was obtained from Guangdong Global Pharmaceutical Co., Ltd (Chinese drug approval numbers: Z10930036, China National Pharmaceutical Group). Blood glucose levels were measured using the ACCU-CHEK Full Activity Glucometer (Roche Diagnostics, Germany).

### 2.2 Experimental animals and grouping

Throughout the experiment, key indicators of the health status of SD rats, including coat condition (hair color), daily food and water intake, excretion, and general activity, were monitored daily. The SD rats were 6–8 weeks old and weighed 250–450 g. Body weight and food intake were recorded weekly, and energy intake was subsequently calculated. A total of 60 SD rats (30 males and 30 females) were procured from Shanghai Slake Experimental Animal Co., Ltd. (license number: SYXK [Shanghai] 2022-0012). All animals were housed in specific pathogen-free (SPF) conditions, maintained on a 12-h light/dark cycle, with a controlled environment of 45% to 65% relative humidity and a temperature of 19–21°C. Following a 1-week acclimatization period, the rats were randomly assigned to the following six groups (*n* = 10): a control group, a model group (HFD), a self-healing group (HFD), a metformin treatment group, a Yupingfeng treatment group, and a combined Yupingfeng and metformin treatment group.

### 2.3 Modelling of type 2 diabetes

Yupingfeng powder solution: The Yupingfeng powder solution was prepared by thoroughly mixing 100 mesh micro-powdered Yupingfeng with distilled water. Based on preliminary testing, the optimal powder-to-water ratio was found to be 1:6, yielding a final concentration of 0.167 g/mL. The metformin aqueous solution was prepared using metformin hydrochloride tablets dissolved in distilled water.

Diet and diabetes model induction: During the modeling period, all experimental groups except the blank control group were fed a high-fat, high-sugar diet along with 5% glucose solution as drinking water. The blank control group received a standard diet and normal drinking water. After 6 to 8 weeks of feeding, all groups, except the blank control group, received an intraperitoneal injection of 2% STZ at a dose of 40 mg/kg body weight. The blank control group received an equivalent volume of sodium citrate buffer as a vehicle control. Three days post-injection, rats were fasted for 12 h to induce mild dehydration prior to glucose testing. Fasting blood glucose levels were measured using tail blood (tail vein sampling). A fasting blood glucose concentration ≥16.7 mmol/L indicated a successful diabetes model induction. Rats that did not meet this threshold received a second STZ injection using the same dose, followed by another fasting blood glucose measurement 3 days later. Where necessary, a third injection was administered. Rats that failed to reach the target blood glucose level after three STZ injections were classified as unresponsive to model induction.

### 2.4 Experimental group treatment

The control group of SD rats consistently received a standard diet, maintaining a normal blood glucose range between 4.77 and 8.27 mmol/L. All the experimental groups were fed an HFD during the initial 8 weeks. By the fourth week, their blood glucose levels exceeded the upper limit of the normal range and remained elevated through the eighth week, signifying successful diabetic model induction.

Starting from the eighth week, all groups resumed access to regular drinking water. The model group continued to receive an HFD throughout the experiment, while the self-healing group and treatment groups were transitioned to a standard diet.

### 2.5 Blood glucose testing

Blood glucose levels were assessed prior to the start of the experiment and subsequently at the end of weeks 1 through 8. The feeding regimen for SD rats were as follows: the control group received a standard diet throughout the study, the model group consistently received an HFD, whereas the self-healing, metformin treatment, Yupingfeng powder treatment, and metformin and Yupingfeng powder combined treatment groups received an HFD during the modeling phase followed by a standard diet during the treatment phase. For the experimental groups, blood glucose levels were measured before the initiation of treatment and at weeks 1 through 7 of the treatment periods. All blood glucose measurements were recorded and saved for subsequent analysis.

### 2.6 Determination of fasting insulin in SD rats

Using procedures of enzyme-linked immunosorbent assay (ELISA), standard solutions were serially diluted, and wells were designated as blank, standard, or sample wells. For the standard wells, 50 μL of each standard solution was accurately added. For the sample wells, 40 μL of sample diluent was added followed by 10 μL of the test sample. The plate was then sealed with a sealing film and incubated at 37°C for 30 min. After incubation, the sealing film was carefully removed. The liquid in each well was discarded, and the wells were blotted dry. Each well was then filled with washing buffer and allowed to stand for 30 s, after which the buffer was discarded. The washing step was repeated five times, followed by blotting to dry the plate. Next, 50 μL of enzyme reagent was added to each well except the blank wells. The plate underwent another round of incubation and washing as described above. Then, 50 μL of Color Reagent A was added to each well, followed by 50 μL of Color Reagent B. The plate was gently oscillated (shaken) to mix and incubated at 37°C in the dark for 15 min. Then, 50 μL of stop solution was added to each well to terminate the reaction.

The absorbance or optical density (OD) value of each well was measured at 450 nm using a microplate reader, with the blank well used to zero the instrument. All measurements were completed within 15 min of adding the stop solution. The insulin concentration was calculated using the standard curve formula: *Y* = 0.03073 * *X* + 0.1709 (*R* ^2^ = 0.9564), where *Y* is the OD value and *X* is the insulin concentration.

### 2.7 Determination of triglycerides

Standard solutions were prepared by serial dilution to obtain concentrations of 24, 16, 8, 4, and 2 nmol/L. Wells were designated as blank, standard, or sample wells. Into each standard, 100 μL of the corresponding standard solution was added. For the sample wells, 50 μL of the appropriately diluted test samples was added. The plate was sealed with a sealing film and incubated at 37°C for 30 min. After incubation, the sealing film was carefully removed, the wells were emptied, and the residual liquid was blotted dry. Each well was then filled with wash buffer, allowed to stand for 30 s, and the buffer was discarded. This washing step was repeated five times, and the plate was gently blotted dry. Subsequently, 50 μL of enzyme reagent was added to each well, except the blank wells. Following another incubation and washing step as described, 50 μL of Color Reagent A was added to each well, followed by 50 μL of Color Reagent B. The plate was gently agitated to mix and incubated at 37°C in the dark for 15 min. Then, 50 μL of stop solution was added to each well to terminate the reaction.

The absorbance or OD value of each well was measured at 450 nm using a microplate reader, with the blank well used to zero the instrument. All measurements were completed within 15 min of adding the stop solution. TG concentrations were calculated based on the standard curve, using the calibration value of *C* = 2.70 mmol/L.


$$TG\;mmol\;L=\frac{\mathrm{A(test)-A(control)}}{\mathrm{A(standard)-A(control)}}\times C$$

### 2.8 Determination of monoamine oxidase, liver, and kidney indicators after treatment

Liver and kidney tissue sample preparation: Approximately 0.1 g of liver or kidney tissue samples was separately homogenized in 1 mL of extraction solution I on ice. The homogenate was centrifuged at 1,000 × *g* for 10 min at 4°C. The supernatant was carefully collected and transferred to a pre-cooled centrifuged tube, followed by a second centrifugation at 1,000 × *g* for 30 min at 4°C. The resulting supernatant was discarded, and the pellet was resuspended in 1 mL of cold extraction solution II. After thorough mixing by gentle vortexing, the sample was centrifuged at 1,000 × *g* for 40 min at 4°C. The final pellet was resuspended in 1 mL of assay reagent, mixed well, and kept on ice for subsequent analysis.

Serum collection and preparation: Freshly collected blood samples were immediately incubated at a 37°C for 30 min to promote coagulation. Subsequently, the samples were centrifuged at 3,000 rpm for 10 min at 4°C. The serum (supernatant) was carefully transferred to a sterile EP tube, ensuring that the red blood cell layer was not disturbed. The serum was used for subsequent analyses, with precautions taken to avoid repeated freeze–thaw cycles. Each sample was labeled with the respective rat identifiers and stored at −20°C until use.

### 2.9 Observation of duodenal and colonic pathology

A total of 60 mice were included in this study. Surgically removed duodenal and colonic tissue samples were trimmed to approximately 0.6 cm^3^ and fixed in 4% paraformaldehyde solution for 3 days. The samples were then dehydrated through a graded ethanol series, cleared in xylene, and embedded in paraffin wax. The resulting paraffin blocks were stored at 4°C for subsequent histological analysis. For histological examination, 5-µm-thick sections were cut and subjected to hematoxylin and eosin (H&E) staining. The sections were dewaxed in xylene, rehydrated through a descending alcohol gradient, stained with hematoxylin followed by eosin, dehydrated again through an ascending alcohol series, cleared in xylene, and mounted using neutral resin.

### 2.10 Statistical analysis

The data are presented as mean ± standard deviation (SD). Statistical analyses were performed using SPSS version 21.0 software. Inter-group comparisons were performed using one-way analysis of variance, followed by pairwise comparison using Student Newman Keuls (SNK) *post-hoc* test. A *p*-value of less than 0.05 (*p* < 0.05) was considered statistically significant.

## 3 Results

### 3.1 Effects of high-fat diet on food intake, water intake, and body weight of SD rats

The initial body weight of SD rats was approximately 150 g, with no significant differences among the groups. Rats in the HFD group exhibited a rapid increase in body weight during the initial phase, peaking at the fourth week, followed by a gradual decline. In contrast, the control group showed a steady and continuous weight gain throughout the study period. The HFD group also demonstrated a marked and sustained increase in both food and water intake, and these changes were highly significant (*p*< 0.01). Similarly, the control group also experienced increased food and water intake corresponding with weight gain, which was statistically significant (*p*< 0.05). These are presented in **Table 1**.

**Table 1:** Changes in food intake, water intake, and body weight of SD rats in the control group and the diabetes model group.

Group		Food ration (g/day)		Water intake (g/day)		Weight (g)	
		Fourth weekend	Eighth weekend	Fourth weekend	Eighth weekend	Fourth weekend	Eighth weekend
Control-G	Male	124.85 ± 15.64	158.95 ± 8.62	265.11 ± 33.24	325.33 ± 28.61	392.12 ± 33.83	469.72 ± 50.45*
	Female	112.45 ± 12.82	134.25 ± 7.84	162.51 ± 18.52	260.91 ± 34.21	252.66 ± 24.55	294.93 ± 48.37*
Model-G	Male	126.75 ± 22.34	254.23 ± 21.66	550.37 ± 46.92	929.38 ± 80.34	411.59 ± 47.72	350.93 ± 43.72***
	Female	106.36 ± 8.66	228.59 ± 15.19	366.54 ± 37.94	649.31 ± 55.62	248.51 ± 19.68	217.83 ± 39.32***

* and *** represent significant differences of mean values.

### 3.2 Effects of high-fat diet on blood glucose levels in SD rats

**Figure 1:**
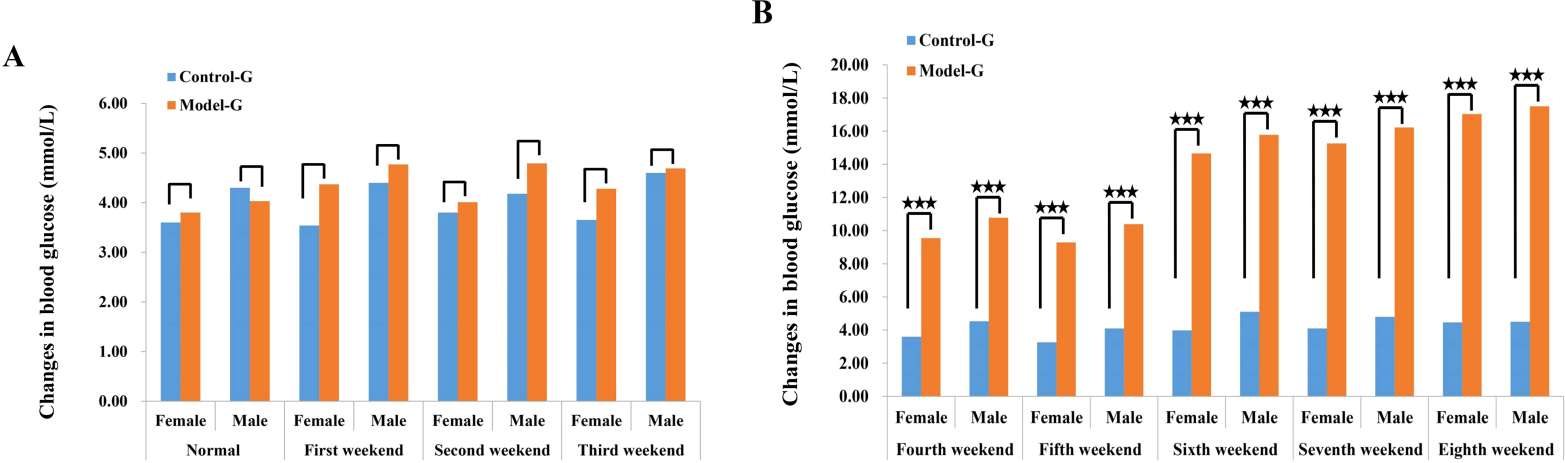
Changes in blood glucose of SD rats in the control group and the model group. **(A)** Blood sugar concentration in female SD rats 3 weeks before modeling. **(B)** The blood glucose concentration of female SD rats between the fourth and eighth week of membrane production. The three five-pointed stars represent significant differences of mean values.

At the end of the 8th week, prior to modeling, male and female SD rats in the HFD group exhibited elevated blood glucose levels of 17.03 and 17.50 mmol/L, respectively. In contrast, blood glucose levels in the normal diet (control) group remained within the normal range, measuring 4.50 mmol/L in males and 4.46 mmol/L in females. Following 8 weeks of HFD feeding, rats in the model group showed a significant increase in blood glucose levels, while those in the control group maintained stable blood glucose levels of approximately 4.5 mmol/L. In the model group, blood glucose began to rise significantly from the fourth week onward, steadily increasing from 4 mmol/L to a peak of approximately 17 mmol/L. Additionally, the data suggest a consistent trend in both groups, where male rats exhibited slightly higher blood glucose levels than females, as illustrated in **Figure 1**.

### 3.3 Detection of key biochemical indicators in the modeling stage

**Figure 2:**
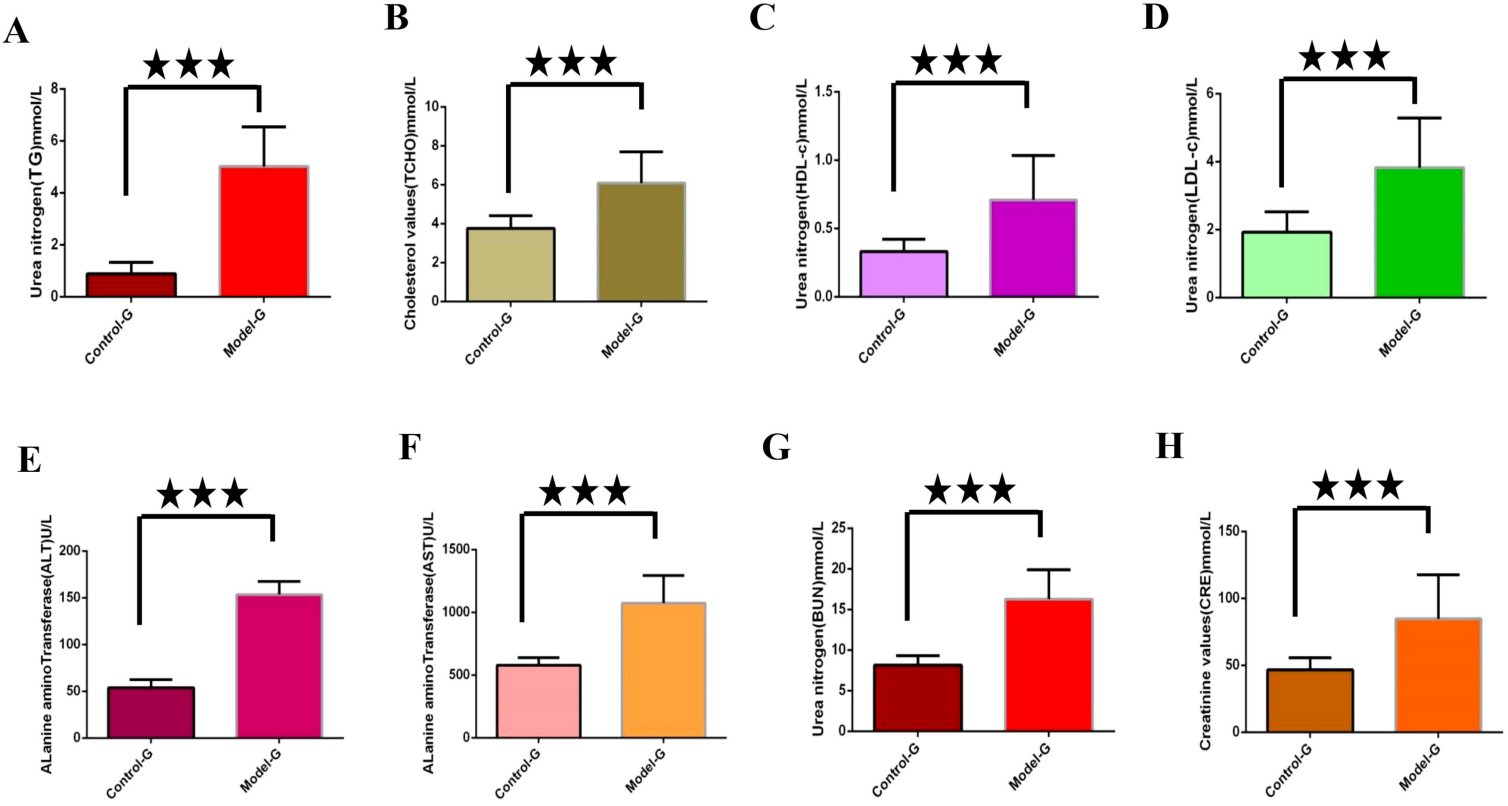
Detection of key biochemical indicators in the modeling stage. **(A)** through **(H)** are control group and model group indicator detection. **(A)** Triglycerides (TG). **(B)** Total cholesterol (TCHO). **(C)** High density lipoprotein cholesterol (HDL-c); **(D)** Low density lipoprotein cholesterol (LDL-c); **(E)** Alanine aminotransferase (ALT). **(F)** Alkaline transaminase (AST). **(G)** Urea nitrogen (BUN). **(H)** Creatinine (CRE). The three five-pointed stars represent significant differences of mean values.

Lipid metabolism markers such as TG, TCHO, LDL-c and high-density lipoprotein cholesterol (HDL-c) were significantly elevated in the model group compared to the control group (*p*< 0.01), as illustrated in **Figures 2A–D**. Additionally, levels of ALT, alkaline transaminase (AST), blood urea nitrogen (BUN), and creatinine (CRE) were significantly increased in the model group relative to the control group (*p*< 0.01), as illustrated in **Figures 2E–H**.

### 3.4 Fasting insulin levels during treatment

**Figure 3:**
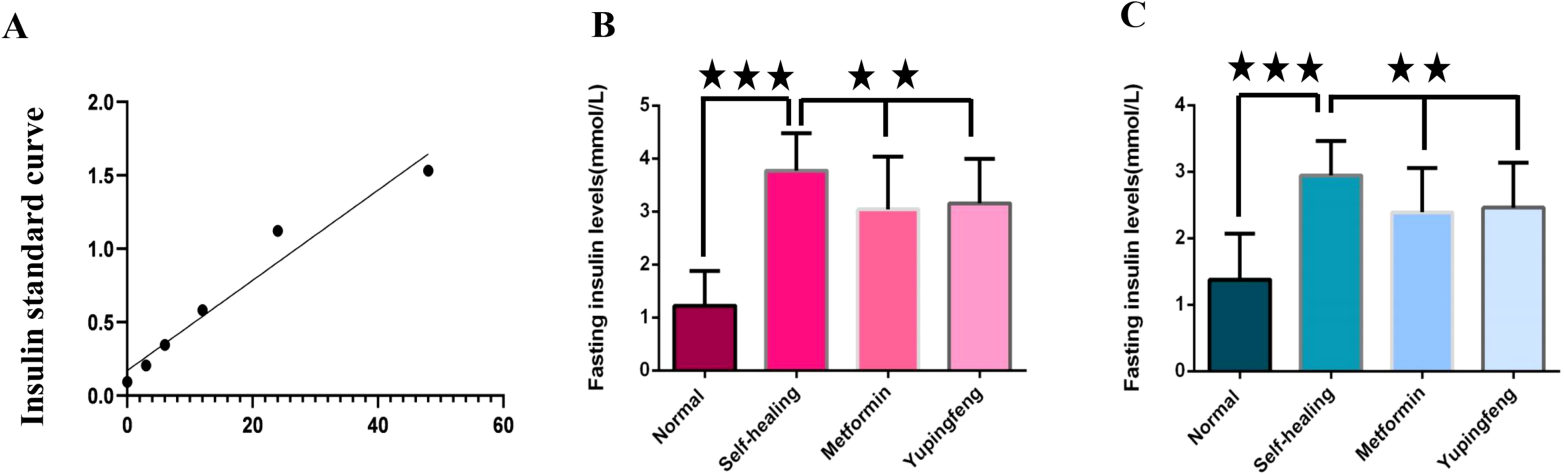
Fasting insulin levels during treatment. **(A)** Fasting insulin standard curve. **(B)** Fasting insulin levels in different groups during the third week of treatment. **(C)** Fasting insulin levels in different groups during the seventh week of treatment.

FINS levels were assessed during the third and seventh weeks of drug treatment. The results indicated a significant reduction in insulin levels in both Yupingfeng and metformin treatment groups, suggesting a trend toward recovery (*p*< 0.05). On the third week, insulin levels decreased from 3.77 to 3.15 mmol/L in the Yupingfeng group and to 3.04 mmol/L in the metformin group. By the seventh week, the levels further declined from 2.94 to 2.39 and 2.47 mmol/L, respectively. These findings are presented in **Table 2** and illustrated in **Figure 3**.

**Table 2:** Fasting insulin levels during treatment.

Group	Control-G	Self-healing-G	Metformin-G	Yupingfeng-G	Met-Yup-G
Third week FINS (pmol/L)	1.22 ± 0.68	3.77 ± 0.75*	3.04 ± 1.09**	3.15 ± 0.74**	2.89 ± 0.88***
Seventh week FINS (pmol/L)	1.37 ± 0.65	2.94 ± 0.53*	2.39 ± 0.61**	2.47 ± 0.52**	2.25 ± 0.77**

*, **, and *** represent significant differences of mean values.

### 3.5 Changes in blood glucose levels and insulin resistance index during treatment

At the conclusion of the treatment period, blood glucose levels for Yupingfeng, metformin, and combination (metformin + Yupingfeng) groups of SD rats in the model group were 17.41, 18.59, and 17.43 mmol/L, respectively. In contrast, rats in the normal diet (control) group maintained normal glucose levels at approximately 4.48 mmol/L. After 7 weeks of treatment, the blood glucose levels in the Yupingfeng, metformin, and the combination therapy (metformin + Yupingfeng) groups showed significant reductions (*p*< 0.01): from 19.52 to 14.99 mmol/L, from 19.32 to 13.27 mmol/L, and from 19.53 to 11.62 mmol/L, respectively. However, the self-healing group did not exhibit a significant decrease in blood glucose levels (*p* > 0.01), with high levels remaining at 17.61 mmol/L, as presented in **Table 3**.

**Table 3:** Changes of blood glucose of SD rats in the model treatment group.

Group	Normal	First weekend	Third weekend	Fifth weekend	Seventh weekend
Control-G	4.48 ± 0.02	4.15 ± 0.28	3.93 ± 0.61	4.65 ± 0.55	4.63 ± 0.63
Self-healing-G	19.19 ± 1.88	17.35 ± 1.71	17.65 ± 1.29	18.35 ± 0.88	17.61 ± 1.79*
Metformin-G	18.59 ± 1.13	19.32 ± 1.25	18.17 ± 1.35	17.48 ± 1.56	13.27 ± 1.59**
Yupingfeng-G	17.41 ± 1.67	19.52 ± 3.76	17.01 ± 2.22	16.64 ± 3.24	14.99 ± 3.41**
Met-Yup-G	17.43 ± 3.25	19.53 ± 3.41	16.94 ± 3.23	15.05 ± 2.58	11.62 ± 1.34***

*, **, and *** represent significant differences of mean values.

During the first 7 weeks of treatment, there was no significant change in the insulin resistance index in the self-healing group (*p* > 0.01). In contrast, significant reductions were observed in the treatment groups. In the Yupingfeng treatment group, the index decreased from 5.97 to 4.83 mmol/L; in the metformin group, it decreased from 5.79 to 4.36 mmol/L; and in the combined metformin and Yupingfeng (met-yup) group, it decreased from 5.82 to 4.01 mmol/L. All reductions in the treatment groups were statistically significant (*p*< 0.01), as presented in **Table 4**.

**Table 4:** Insulin resistance index in rats (HOMA-IR).

Group	First weekend	Third weekend	Fifth weekend	Seventh weekend
Control-G	3.83 ± 0.32	3.91 ± 0.91	4.11 ± 0.51	4.09 ± 0.96
Self-healing-G	5.51 ± 0.65	5.69 ± 0.55	5.49 ± 1.32	6.38 ± 1.58*
Metformin-G	5.79 ± 1.21	5.39 ± 1.16	5.02 ± 1.71	4.36 ± 1.83*
Yupingfeng-G	5.97 ± 1.37	5.42 ± 1.43	5.24 ± 1.67	4.83 ± 1.55***
Met-Yup-G	5.82 ± 1.71	5.32 ± 1.04	5.08 ± 0.54	4.01 ± 0.81**

*, **, and *** represent significant differences of mean values.

### 3.6 Changes MAO resistance index during treatment

At 15 and 30 min after the initiation of treatment, monoamine oxidase (MAO) levels did not change significantly in the self-healing group (*p*< 0.01). In contrast, significant reductions were observed in all treatment groups. In the Yupingfeng treatment group, MAO levels decreased from 0.47 to 0.39 mmol/L; in the metformin group, from 0.39 to 0.32 mmol/L; and in the combined metformin and Yupingfeng (met-yup) group, from 0.41 to 0.34 mmol/L, These reductions were statistically significant (*p*< 0.01), as presented in **Table 5**.

**Table 5:** Monoamine oxidase (MAO) changes.

Group (MAO)	15 min		20 min		30 min	
	Best fit	Average	Best fit	Average	Best fit	Average
Control-G	0.2419	0.2761	0.2188	0.2521	0.1972	0.2289
Self-healing-G	0.3363	0.3039*	0.3033	0.2771*	0.2736	0.2518*
Metformin-G	0.3594	0.3976**	0.3261	0.3612**	0.2956	0.3283**
Yupingfeng-G	0.4211	0.4733***	0.3819	0.4311***	0.3454	0.3918***
Met-Yup-G	0.3836	0.4156**	0.3511	0.3797**	0.3195	0.3455**

*, **, and *** represent significant differences of mean values.

### 3.7 Metabolic liver and kidney indicators after treatment

**Figure 4:**
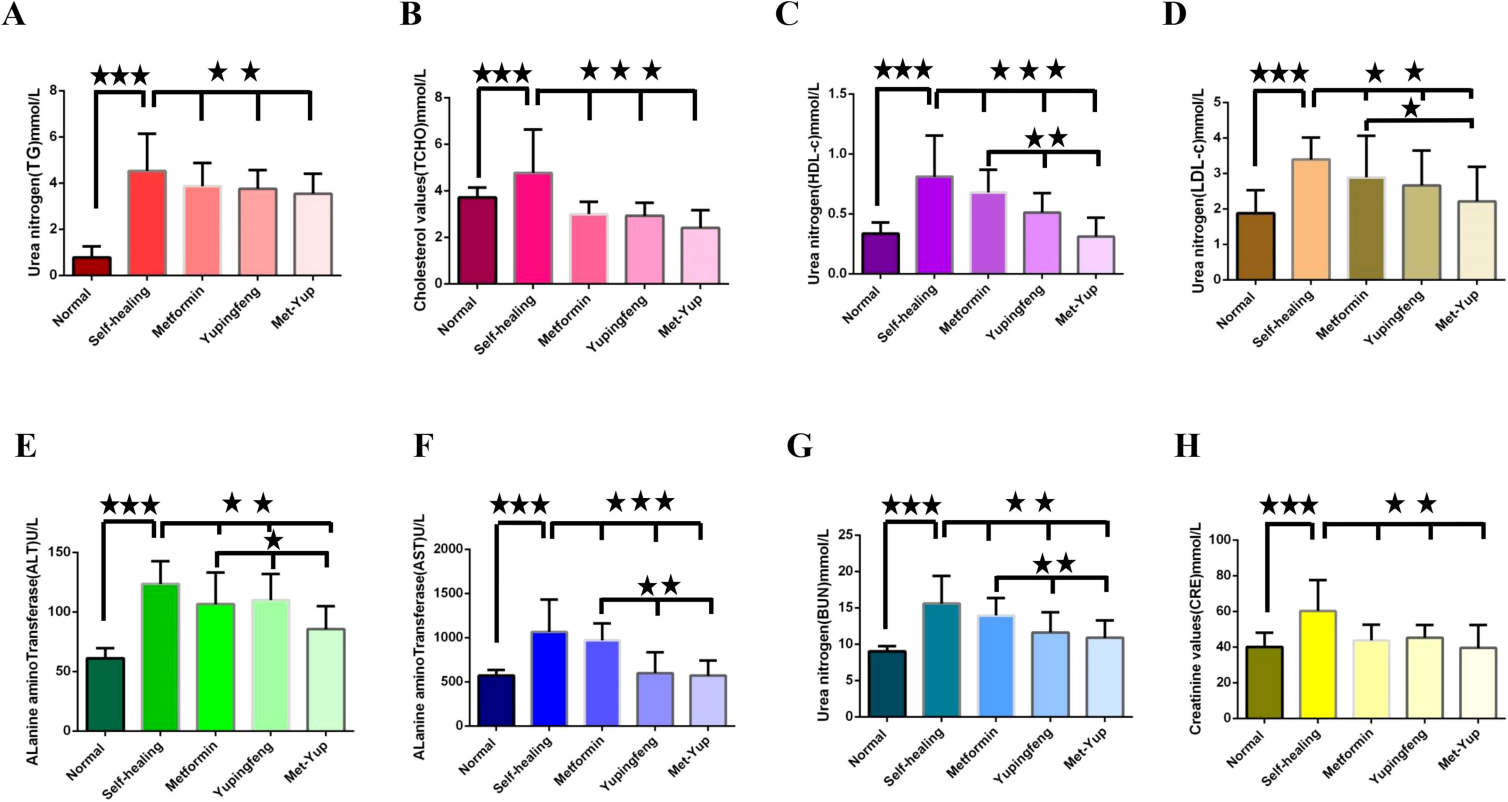
The influence of blood glucose on the rise of biochemical indexes. **(A–H)** are the test indicators for different groups of treatment. **(A)** Triglycerides (TG). **(B)** Total cholesterol (TCHO). **(C)** High density lipoprotein cholesterol (HDL-c); **(D)** Low density lipoprotein cholesterol (LDL-c); . **(E)** Alanine aminotransferase (ALT). **(F)** Alkaline transaminase (AST). **(G)** Urea nitrogen (BUN). **(H)** Creatinine (CRE). The three five-pointed stars represent significant differences of mean values.

In SD rats, indicators of lipid metabolism—including TG, TCHO, LDL-c, and HDL-c—were significantly reduced in the Yupingfeng, metformin, and combined metformin–Yupingfeng (met-yup) treatment groups compared to the self-healing group (*p*< 0.01). However, these values remained higher than those in the control group. Notably, HDL-c and LDL-c levels in the combination treatment group were significantly lower than those in the single drug treatment groups (*p*< 0.05), as shown in **Figures 4A–D**.

Important physiological indicators of liver and kidney function such as ALT, AST, BUN, and CRE levels were elevated in the self-healing group. These levels were significantly low in the Yupingfeng, metformin, and the met-yup treatment groups (*p*< 0.01), although they remained higher than the control group. In addition, ALT, AST and BUN levels in the combination treatment group were significantly lower compared to the single drug group (*p*< 0.05). as shown in **Figures 4E–H**.

### 3.8 Histological observation of duodenal pathology

**Figure 5:**
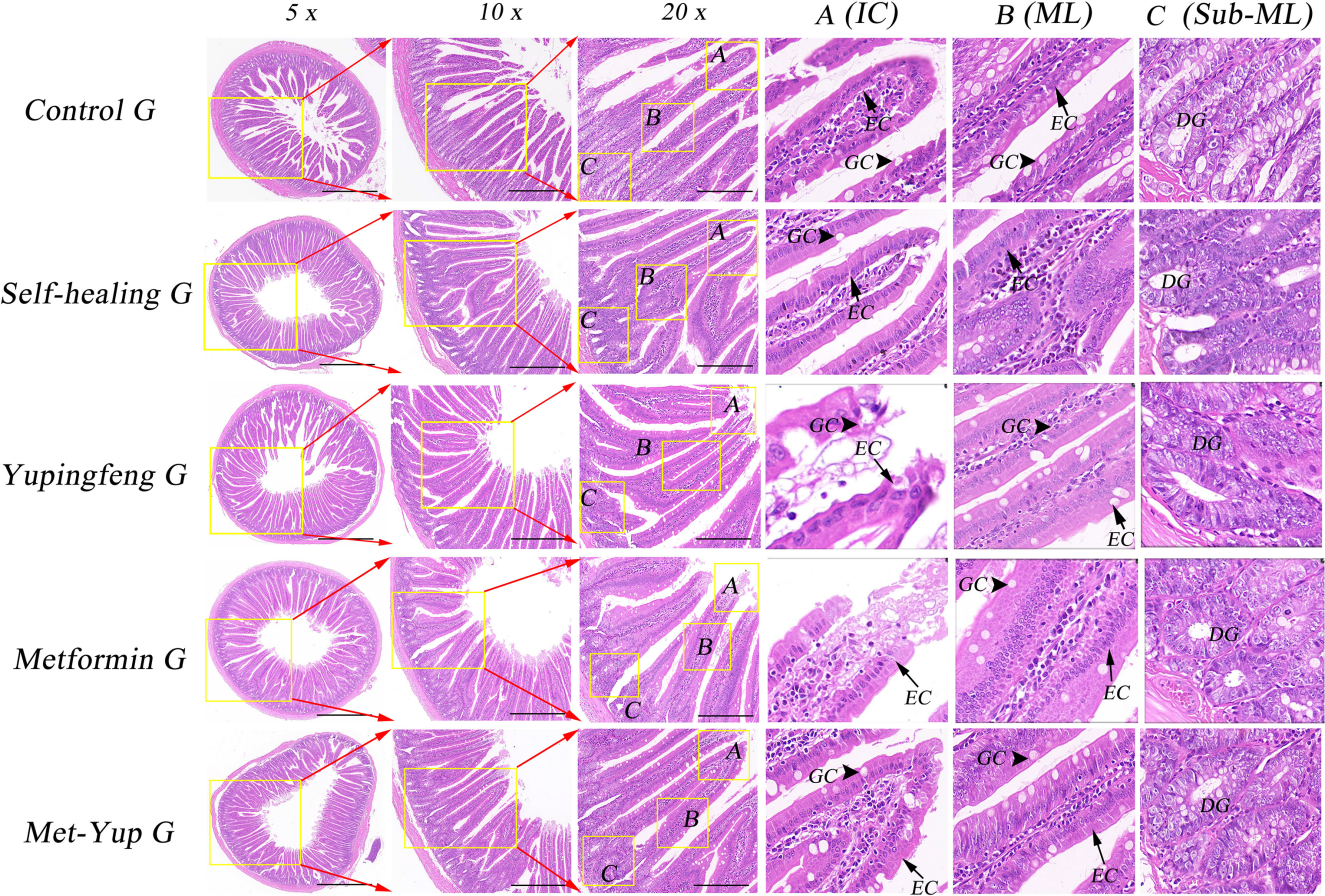
H&E staining of duodenum histopathology. 5× is a 50× enlarged image, 10× is a 100× enlarged image, and 20× is a 200× enlarged image. **(A–C)** are enlarged images of local intestinal tissues. EC, epithelial cells; GC, goblet cell; DG, duodenal gland.

Histological examination of the duodenum revealed notable pathological changes in the model group, including shortened mucosal villi, multilayered epithelial cells, reduced number of goblet (cup) cells, and marked infiltration of inflammatory cells. In the self-healing group, partial recovery of intestinal mucosa was observed, characterized by predominantly monolayer epithelial cells, a reduced number of goblet cells, and mild inflammatory cell infiltration in the lamina propria. In contrast, the duodenal mucosa in the combination treatment group showed near-complete restoration, with well-organized monolayer epithelial cells, a normal number of goblet cells, and no observable inflammatory cell infiltration, as shown in **Figure 5**.

### 3.9 Histological observation of colonic mucosa pathology

**Figure 6:**
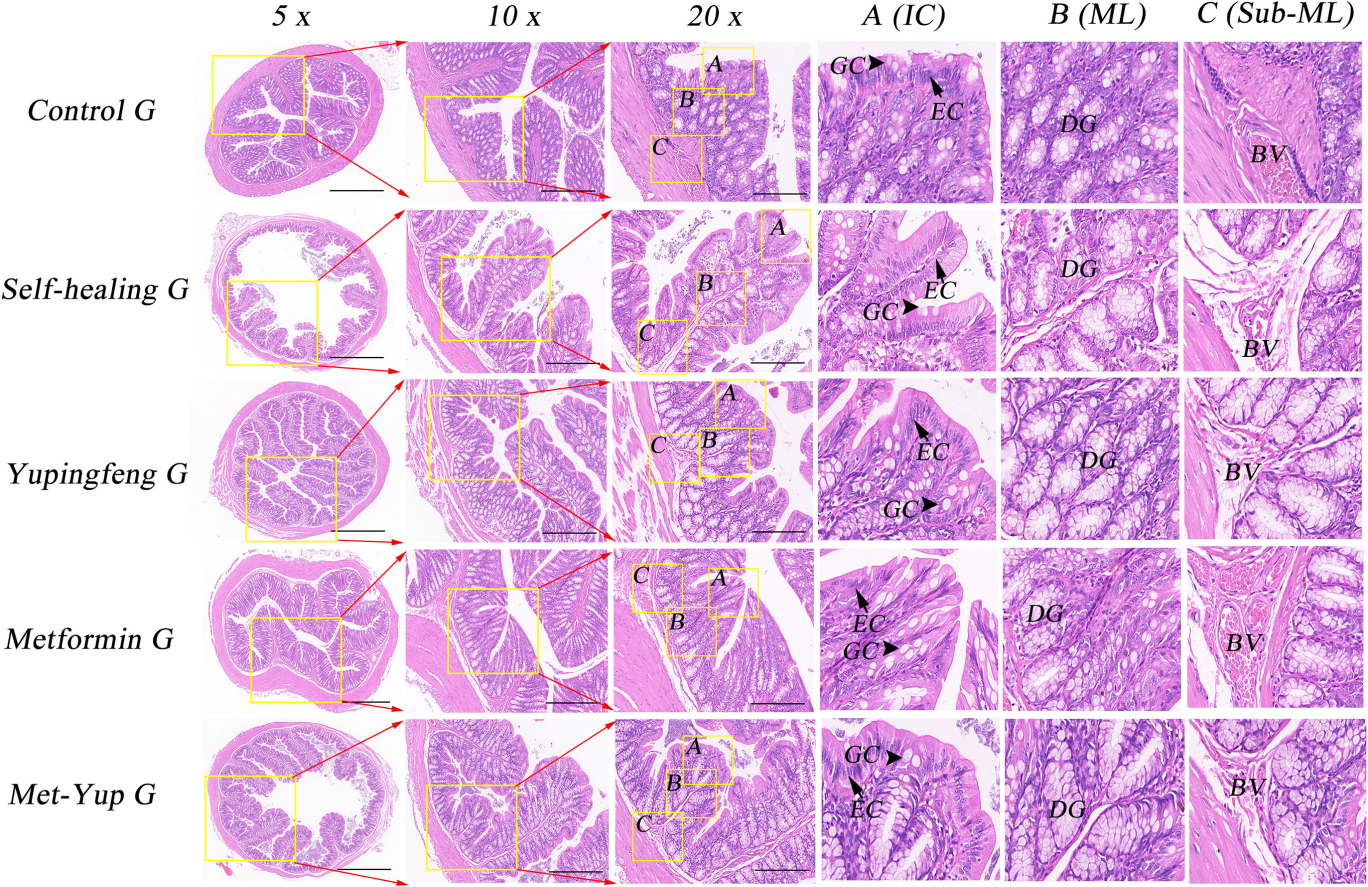
H&E staining of colonic mucosa. 5× is a 50× enlarged image, 10× is a 100× enlarged image, and 20× is a 200× enlarged image. A-C are enlarged images of local intestinal tissues. EC, epithelial cells; GC, goblet cell; DG, duodenal gland.

Histopathological analysis of the colonic mucosa in the HFD group revealed several notable changes including shortened mucosal structures, multilayered epithelial cells, reduced number of goblet cells, and significant infiltration of inflammatory cells. In the self-healing group, partial mucosal recovery was observed, characterized by predominantly single-layered epithelial cells, a reduced number of goblet cells, and mild inflammatory cell infiltration in the lamina propria. However, the combination treatment group exhibited significant improvement. The colonic mucosa essentially appeared nearly normal, with well-organized single-layered epithelial cells, a restored number of goblet cells, and absence of inflammatory cell infiltration, as illustrated in **Figure 6**.

## 4 Discussion

Dietary restrictions in patients with T2D can significantly reduce body weight, lower insulin resistance index and baseline glycosylated hemoglobin (HbA1c) levels, and improve overall metabolic function (18). Our experimental data also confirmed these findings. Prior to modeling SD rats, at the end of week 8, blood glucose levels in the HFD group were 17.03 mmol/L (male) and 15.50 mmol/L (female), compared to 4.50 (male) mmol/L and 4.46 (female) mmol/L in the normal diet (control) group. Recent data from Endocrinology departments across the United States in 2023 further support the correlation between stable blood glucose levels and healthier metabolic status, as reflected in HbA1c measurements (19). In our study, hematological evaluations indicated that lipid metabolism markers, specifically TG, TCHO, LDL-c, and HDL-c, as well as liver and kidney function indicators, viz, ALT, BUN, and CRE, were significantly elevated in the HFD (model) group compared to the control group. Key contributors to cardiovascular disease in patients with T2D include hyperglycemia, dyslipidemia, obesity, insulin resistance, and chronic inflammation. Notably, the activation of pro-inflammatory mechanisms is closely linked to metabolic toxicity and insulin resistance, which are often exacerbated by dysbiosis within the gut microbiota (20). Recent studies have reported no significant distinctions in HbA1c or short-term blood glucose status, such as average blood glucose levels, with similar fluctuations observed between daytime and nighttime glucose levels (21). Consequently, the administration of an HFD to obese rats resulted in significant differences in lipid metabolism, liver function, and kidney biomarkers, aligning with the pathophysiological characteristics of T2D.

TCM has a long history of use in China for the treatment of type 2 diabetes mellitus (T2DM), and the diverse pharmacological effects associated with Yupingfeng have recently attracted increasing scientific interest. In an anti-inflammatory study evaluating the effects of Yupingfeng powder on colitis, the body weight of colitis-afflicted mice significantly decreased by 10%, 12%, 14%, and 17% (*p*< 0.01) on the first, third, fifth, and seventh day of administration, respectively (22). In our experiment, a significant gradual decline in FINS levels was observed during the third and seventh weeks of treatment with Yupingfeng and metformin, although the levels remained higher than in the control group. Hosseini et al. (23) studied the effects of curcumin-piperine supplementation on blood glucose and lipid profiles in patients with T2DM. After 12 weeks of intervention, the curcumin-piperine group exhibited significant reduction in the levels of TG and fasting blood glucose compared to the placebo group, with marginal reduction in C-reactive protein (CRP) levels. Our analysis further showed that from the first week, rats receiving Yupingfeng and metformin exhibited significantly decreased blood glucose concentrations and a reduction in the insulin resistance index. In summary, these findings suggest that the combination of Yupingfeng and metformin effectively reduced FINS levels, blood glucose, and insulin resistance in rats with HFD-induced T2D. Therefore, the results of this experiment indicate that Yupingfeng may also have therapeutic potential as a complimentary treatment for T2D.

Metformin remains the most commonly prescribed drug for managing blood glucose levels in patients with T2DM. Clinical studies have shown that the combination of metformin with SGLT2 inhibitors can further reduce blood glucose concentrations before and after fasting in patients with clinical T2DM, as well as increase glucose-to-creatinine ratio and promote weight loss (16). Comparative transcriptomic analyses have revealed notable differences between the TCM Shenqi compound (SQC) and metformin. Specifically, a total of 962 differentially expressed genes (DEGs) were identified between the SQC and metformin treatment groups, with significant associations to pathways involved in sensory perception of chemical stimuli, NADH dehydrogenase (ubiquinone) activity, and the positive regulation of fatty acid metabolism processes (24). This experimental hematological evaluation revealed a significant reduction in the levels of lipid metabolism markers such as TG, TCHO, LDL-c, and HDL-c in both the metformin and Yupingfeng treatment group compared to the self-healing group. In addition, monoamine oxidase (MAO) levels, measured at 15, 20, and 30 min, showed downward trend across all groups, with the combination treatment group showing the highest initial levels and the most marked decline over time. It is worth noting that other studies have suggested that probiotic fermented milk supplementation may offer benefits in improving glucose and lipid metabolism, as well as reducing inflammation in patients with T2DM (25). Therefore, these findings support the view that pharmacological interventions for T2D can induce a wide range of physiological and metabolic changes in the body, highlighting the complexity and potential of combination therapies in diabetes management.

Hepatorenal metabolism function is a critical indicator for evaluating therapeutic efficacy of treatments for T2DM (26). In our study, hematological assessments indicated that markers of hepatic and renal function, such as ALT, AST, BUN, and CRE, were significantly lower in both metformin and Yupingfeng treatment groups compared to the self-healing group, indicating improved metabolic outcomes. Clinical trials have also demonstrated that Yupingfeng (Jade screens) can counteract viral inflammatory responses by modulating key metabolic pathways involving pyruvate, valine, leucine, and isoleucine (27). However, the effects of Yupingfeng appear to vary by condition. For example, in children with asthma, treatment led to a significant reduction in serum IgE levels, but no significant changes in the levels of blood glucose or lipid metabolism (12). Additionally, earlier studies have shown that Yupingfeng can enhance humoral and cellular immune functions of immunosuppressed mice, especially to prolong hypersensitivity responses, as well as regulate T-helper (Th) cells and T lymphocyte subpopulation (28). These research findings offer theoretical and practical significance into the potentials of Chinese medicine, such as Yupingfeng in supporting blood glucose regulation and broader management of diabetes.

At present, a key focus of the current research is the causal relationship between obesity, enteritis, and T2DM, specifically whether obesity-induced T2DM leads to intestinal inflammation, or whether enteritis exacerbates obesity and accelerates the onset of T2DM. Studies have also found that certain natural compounds, such as anthocyanins, offer promising therapeutic effects by increasing the uptake and utilization of glucose in streptoureamycin-induced diabetic rats and mice, and inhibit intestinal α-glucosidase and pancreatic α-amylase. Similarly, soybean flavonoids, through the metabolism of glycosylated forms such as soybonide by gut bacteria, exhibit the potential for treating pathological and physiological abnormalities associated with T2D (29). We examined the self-healing capacity of the duodenum and colon in diabetic rats, specifically focusing on the groups treated with a combination of Yupingfeng and metformin. Over time, it was observed that the mucosal structure exhibited notable restoration including reformation of a monolayer of epithelial cells, a marked increase in goblet cell numbers, and a significant improvement in the inflammatory response indicating effective repair of intestinal integrity and mitigation of inflammation.

There is growing evidence linking T2DM with ulcerative colitis, primarily through shared mechanisms involving immune dysregulation and local inflammatory responses driven by immunosuppressive cells (3). In a rat model of chronic bronchitis, Yupingfeng was shown to significantly alleviate inflammatory symptoms in lung and bronchial tissues, highlighting its immune-modulatory and anti-inflammatory properties (30). Additionally, in mice fed HFD for 6 weeks, elevated expression of pro-inflammatory cytokines in the small intestine and colon at high expression was observed with a pronounced intestinal inflammatory response week (31). These findings support the hypothesis that an HFD can increase intestinal inflammation and epithelial permeability of intestinal mucosa, allowing lipopolysaccharides (LPS) from gut microbiota to enter systemic circulation. This process may trigger metabolic endotoxemia and contribute to the pathogenesis of T2DM (32). In addition, the H&E staining results further confirm that HFD-induced diabetes is associated with inflammation of the duodenum and colon. Importantly, the combined treatment with Yupingfeng and metformin effectively alleviated inflammatory symptoms and promoted mucosal healing.

## 5 Conclusions

We successfully established a T2D model in SD rats using an HFD. Treatment with the combination of Yupingfeng and metformin significantly reduced blood glucose, improved lipid metabolism, and enhanced liver and kidney function. In particular, this combination therapy demonstrated greater efficacy in alleviating intestinal inflammation and improving key metabolic indicators compared to a single drug group. These findings suggest that Yupingfeng holds promising potential as an adjunct therapy in the clinical management of T2D.

## Data Availability

The original contributions presented in the study are included in the article/supplementary material. Further inquiries can be directed to the corresponding author.
